# Evaluation of risk factors of vertebral fracture in Japanese female patients with glucocorticoid-induced osteoporosis

**DOI:** 10.1186/s13018-020-01813-4

**Published:** 2020-07-29

**Authors:** Yu Mori, Takuya Izumiyama, Kazuyoshi Baba, Naoko Mori, Hiroshi Fujii, Tomonori Ishii, Eiji Itoi

**Affiliations:** 1 Department of Orthopaedic Surgery, Tohoku University Graduate School of Medicine, 1-1 Seiryo machi, Aobaku, Sendai, Miyagi 980-8574 Japan; 2 Department of Diagnostic Radiology, Tohoku University Graduate School of Medicine, 1-1 Seiryo machi, Aobaku, Sendai, Miyagi 980-8574 Japan; 3 Department of Hematology and Rheumatology, Tohoku University Graduate School of Medicine, 1-1 Seiryo machi, Aobaku, Sendai, Miyagi 980-8574 Japan

**Keywords:** Glucocorticoid, Osteoporosis, Vertebral fracture, Bone mineral density, Rheumatological disease

## Abstract

**Background:**

Glucocorticoid-induced osteoporosis and vertebral fracture are common complications in patients on glucocorticoid treatment for rheumatological diseases. The present study aimed to identify the risk factors of vertebral fracture in Japanese female patients with glucocorticoid-induced osteoporosis.

**Methods:**

This study included 225 Japanese women with glucocorticoid-induced osteoporosis and 72 patients with postmenopausal osteoporosis. All participants were treated with bisphosphonate or denosumab for osteoporosis with active form of vitamin D for at least 3 years. The differences of clinical parameters, including age, disease duration, body mass index (BMI), bone mineral density (BMD), and the dose and treatment duration of glucocorticoid were assessed between patients with and without vertebral fracture. Multivariate logistic regression analysis was also performed to evaluate the association of vertebral fracture with clinical parameters.

**Results:**

The significant differences related to age, BMD of the hip, disease duration, glucocorticoid treatment duration between patients with and without vertebral fractures were demonstrated. The present study indicated that disease duration, BMI, and the total hip BMD were independent risk factors for vertebral fractures in patients with glucocorticoid-induced osteoporosis.

**Conclusions:**

Prolonged disease duration, low BMI, and low total hip BMD could be risk factors of vertebral fracture in patients on glucocorticoid treatment for rheumatological diseases.

## Key points

- Little is known regarding the risk factors of vertebral fractures in patients with glucocorticoid-induced osteoporosis.
- We determined that longer disease duration, low BMI, and low total hip BMD were independent risk factors of vertebral fractures in patients with glucocorticoid-induced osteoporosis.
- Our study findings would help physicians to be aware that patients with the above risk factors need to be treated as high risk for vertebral fractures and osteoporosis if they are on glucocorticoid for their rheumatological diseases.

## Introduction

Glucocorticoid is still used for the treatment of rheumatological diseases despite the development of targeted treatment agents [1]. Notably, glucocorticoid-induced osteoporosis is the most common form of secondary osteoporosis [2–5]. Glucocorticoid-induced osteoporosis is caused by the combined mechanism of reduced bone formation and increased bone resorption [6–8]. It is estimated that oral intake of glucocorticoid is associated with a 30%–120% increased risk of hip fracture and a two- to threefold increased risk of vertebral fracture compared with patients not using glucocorticoid [9]. Previous studies have reported that the fracture risk of patients on glucocorticoid was related to the dose and duration of glucocorticoid treatment [2, 8, 10]. Nonetheless, the association of glucocorticoid exposure with bone loss and fracture risk remains unclear [7, 10]. A strong association exists between bone mineral density (BMD) and bone strength, but asymptomatic fractures can occur in glucocorticoid users even without apparent bone loss [11]. Several studies on glucocorticoid-induced osteoporosis have failed to identify the correlation between glucocorticoid dose and bone loss [12, 13]. Moreover, the reason for the increased bone fragility of glucocorticoid users remains unclear.

Randomized, controlled clinical trials were performed to assess the effect of bisphosphonate treatment on glucocorticoid-induced osteoporosis. These studies demonstrated that bisphosphonates, such as alendronate, risedronate, and zoledronic acid, could prevent bone loss in glucocorticoid users [14–16]. Several studies have reported the fracture prevention effect of bisphosphonates in patients with glucocorticoid-induced osteoporosis [17, 18]. The effects of denosumab and risedronate treatments in glucocorticoid-induced osteoporosis were compared and revealed a superior effect of denosumab in increasing lumbar and hip BMD [19]. Several studies have demonstrated the fracture prevention effect of teriparatide in patients with glucocorticoid-induced osteoporosis [20, 21]. The results of these clinical trials demonstrated favorable effects of treatment for glucocorticoid-induced osteoporosis. However, in a clinical setting, the prevention of bone loss and vertebral fracture in patients with glucocorticoid-induced osteoporosis is challenging because of complications or low adherence to the guidelines of management and treatment [22, 23].

The present study aimed to identify the risk factors of vertebral fracture in Japanese patients with glucocorticoid-induced osteoporosis on drug treatment for inhibition of bone resorption, comparing with postmenopausal osteoporosis patients. We hypothesized that age, disease duration, body mass index, BMD, and the cumulative glucocorticoid dose could be related to the incidence of vertebral fracture.

## Materials and methods

### Patients

This cross-sectional, retrospective study was approved by the Institutional Review Board of our institute. Informed consent was obtained from all patients before participation in this study, as per the Declaration of Helsinki. The study population included 225 Japanese female patients with glucocorticoid-induced osteoporosis with a history of rheumatological disorders, such as rheumatoid arthritis, systemic lupus erythematosus, scleroderma, dermatomyositis, mixed connective tissue disease, and vasculitis. Patients fulfilled the diagnostic criteria of each rheumatological disease, as well as the Japanese Society of Osteoporosis criteria for the diagnosis of glucocorticoid-induced osteoporosis [24]. Participants who were on glucocorticoid treatment for at least 3 years were included. In addition, 72 Japanese patients with postmenopausal osteoporosis were included as the control group. All these patients fulfilled the Japanese Society of Osteoporosis criteria for the diagnosis of osteoporosis [25]. Patients were enrolled at our institution from April 2016 through March 2020. All patients were on the active form of vitamin D. In addition, participants had been treated with bisphosphonate or denosumab for osteoporosis for at least 3 years. We excluded patients with parathyroid disease, chronic severe renal dysfunction, and malabsorption disease, as well as patients who had been treated with parathyroid hormone and romosozumab. Furthermore, we excluded patients with avascular necrosis of the hip and vertebral fractures caused by high-velocity injuries.

### Clinical variables

General characteristics were recorded in detail, including age, height, bodyweight, and menopause in patients with postmenopausal osteoporosis and glucocorticoid-induced osteoporosis. In addition, the disease condition that necessitated glucocorticoid treatment, disease duration, glucocorticoid treatment duration, and the current and the cumulative doses of glucocorticoid were recorded in patients with glucocorticoid-induced osteoporosis.

### BMD measurements and vertebral fracture assessment

We measured BMD (g/cm^2^) at the AP lumbar spine (vertebrae L2–4) and left hip (total hip and femoral neck) by using dual-energy X-ray absorptiometry (Discovery DXA system; Hologic, Waltham, MA, USA). All procedures were performed according to the manufacturer’s standardized protocols at the time of enrollment. All BMD results were expressed as absolute values (g/cm^2^). Thoracolumbar radiographs were obtained in all patients to detect vertebral fractures, including both painful vertebral fractures and asymptomatic morphologic vertebral fractures at the time of enrollment.

### Statistical analysis

All results were expressed as the mean ± standard deviation. Comparisons between postmenopausal osteoporosis and glucocorticoid-induced osteoporosis groups were performed using the Mann–Whitney *U* test. Mann–Whitney *U* test was performed to identify the differences in clinical parameters, such as age, BMI, and BMD of the lumbar spine, femoral neck, and total hip between patients with and without vertebral fractures in the postmenopausal osteoporosis group. Similarly, the clinical parameters, including age, BMI, disease duration, disease duration subtracted from age, treatment duration of glucocorticoid, present and cumulative doses of glucocorticoid, and BMD of the lumbar spine, femoral neck, and total hip were compared using Mann–Whitney *U* test between patients with and without vertebral fractures in the glucocorticoid-induced osteoporosis group. Fisher’s exact test was also performed to assess the association of menopause status and prevalence of vertebral fractures in patients with glucocorticoid-induced osteoporosis. Statistical models were developed to identify the risk factors of vertebral fractures in patients with glucocorticoid-induced osteoporosis. Multivariate logistic regression analysis was performed, wherein covariates included in the model were explanatory variables that achieved a *p* value of < 0.2 on bivariate analyses. A backward stepwise selection procedure was performed to obtain the final set of variables included in the model. Odds ratio (OR) and 95% confidence interval (CI) were reported for variables. All statistical tests were two-sided, with *p* value of less than 0.05 considered statistically significant. All analyses were performed using JMP version 15 (SAS, Cary, NC, USA).

## Results

Table 1 presents the clinical characteristics of patients with postmenopausal osteoporosis and glucocorticoid-induced osteoporosis. Significant intergroup differences were observed related to age, height, and bodyweight. Notably, the glucocorticoid group had a lower average age and higher height and body weight compared with the postmenopausal osteoporosis group. All parameters of BMD were significantly higher in the glucocorticoid-induced osteoporosis group [lumbar spine: 0.775 (0.118) vs. 0.884 (0.13) g/cm^2^, respectively, *p* < 0.0001; femoral neck: 0.541 (0.12) vs. 0.654 (0.118) g/cm^2^, *p* < 0.0001; total hip: 0.639 (0.114) vs. 0.753 (0.125) g/cm^2^, *p* < 0.0001]. The prevalence of vertebral fractures of thoracic and lumbar spines in the postmenopausal osteoporosis group was 41.7% (30/72), and that in the glucocorticoid-induced osteoporosis group was 27.6% (62/225). In the glucocorticoid-induced osteoporosis group, there was a significant association between menopause status and prevalence of vertebral fracture (*p* < 0.0001). The details of medical conditions that necessitated glucocorticoid therapy are presented in Table 1.

**Table 1:** Clinical characteristics of patients with postmenopausal osteoporosis and glucocorticoid-induced osteoporosis

	Postmenopausal osteoporosis (*n* = 72)	Glucocorticoid-induced osteoporosis (*n* = 225)	*p* value
Premenopausal (%)		144 (64)	
Postmenopausal (%)	72 (100)	81 (36)	
Age (years)	76.4 (8.16)	49.7 (15.6)	< 0.0001***
Height (cm)	148.4 (8.42)	156.5 (7.07)	< 0.0001***
Bodyweight (kg)	48.2 (8.4)	53.3 (11.1)	0.013*
Body mass index	21.9 (3.45)	21.8 (4.08)	0.71
Bone mineral density (g/cm^2^)			
Lumbar spine	0.775 (0.118)	0.884 (0.13)	< 0.0001***
Femoral neck	0.541 (0.12)	0.654 (0.118)	< 0.0001***
Total hip	0.639 (0.114)	0.753 (0.125)	< 0.0001***
Vertebral fracture (%)	30 (41.7)	62 (27.6)	
Medical conditions necessitating glucocorticoid therapy			
Rheumatoid arthritis (%)		57 (25.3)	
Systemic lupus erythematosus (%)		148 (65.8)	
Others (%)		20 (8.9)	
Disease duration (years)		15.9 (10.5)	
Disease duration subtracted from age (years)		33.7 (14.8)	
Glucocorticoid treatment duration (years)		14.4 (10.1)	
Daily prednisone-equivalent dose (mg)		8.1 (3.42)	
Cumulative prednisone-equivalent dose (g)		402.9 (387.6)	
Treatment for osteoporosis			
Bisphosphonate (%)	61 (84.7)	172 (76.4)	
Denosumab (%)	11 (15.3)	53 (22.6)	

Results are expressed as the mean and standard deviation

*p < 0.05

***p < 0.001 by Mann–Whitney *U* test

Table 2 provides the comparison of clinical parameters in patients with and without vertebral fractures in the postmenopausal osteoporosis group. Age was significantly higher in patients with vertebral fractures [72.2 (8.3) vs. 79.7 (6.4) years, *p* = 0.0038]. In contrast, no significant differences related to BMI and BMD were noted between patients with and without vertebral fractures. Table 3 presents the comparison of clinical parameters in patients with and without vertebral fractures in the glucocorticoid-induced osteoporosis group. Age was significantly higher in patients with vertebral fractures [45.7 (14.3) vs. 61.8 (13.1) years, *p* < 0.0001]. Upon comparison of BMD, patients with vertebral fractures had significantly lower values at the femoral neck [0.675 (0.11) vs. 0.589 (0.115) g/cm^2^, *p* < 0.0001] and total hip [0.78 (0.118) vs. 0.671 (0.11) g/cm^2^, *p* < 0.0001]. However, no significant differences were noted related to the BMD of the lumbar spine. Disease duration was significantly longer in patients with vertebral fractures [13.8 (9.52) vs. 22.4 (10.6) years, *p* < 0.0001]. The disease duration subtracted from age was significantly longer in patients with vertebral fractures [31.8 (14.4) vs. 39.4 (14.7) years, *p* = 0.0039]. Glucocorticoid treatment duration was significantly longer than in patients with vertebral fractures [12.1 (10.2) vs. 21.6 (10.4) years, *p* < 0.0001]. However, no significant differences were observed regarding the current and cumulative glucocorticoid doses.

**Table 2:** Comparison of clinical measurements in patients with postmenopausal osteoporosis with and without vertebral fractures

	Without vertebral fractures (*n* = 42)	With vertebral fractures (*n* = 30)	*p* value
Age (years)	72.2 (8.3)	79.7 (6.4)	0.0038**
Body mass index	21.5 (3.8)	22.1 (3.2)	0.47
Bone mineral density (g/cm^2^)			
Lumbar spine	0.767 (0.09)	0.79 (0.13)	0.45
Femoral neck	0.563 (0.12)	0.525 (0.122)	0.64
Total hip	0.671 (0.085)	0.61 (0.128)	0.37

Results are expressed as the mean and standard deviation

**p < 0.01 by Mann–Whitney *U* test

**Table 3:** Comparison of clinical measurements in patients with glucocorticoid-induced osteoporosis with and without vertebral fractures

	Without vertebral fractures (*n* = 163)	With vertebral fractures (*n* = 62)	*p* value
Age (years)	45.7 (14.3)	61.8 (13.1)	< 0.0001***
Body mass index	22.12 (4.13)	20.99 (3.86)	0.18
Bone mineral density (g/cm^2^)			
Lumbar spine	0.89 (0.12)	0.86 (0.148)	0.087
Femoral neck	0.675 (0.11)	0.589 (0.115)	< 0.0001***
Total hip	0.78 (0.118)	0.671 (0.11)	< 0.0001***
Disease duration (years)	13.8 (9.52)	22.4 (10.6)	< 0.0001***
Disease duration subtracted from age (years)	31.8 (14.4)	39.4 (14.7)	0.0039**
GC treatment duration (years)	12.1 (10.2)	21.6 (10.4)	< 0.0001***
Present GC dose (mg)	8.06 (3.51)	8.24 (3.14)	0.57
Cumulative GC dose (g)	374.1 (347.6)	490.1 (482.6)	0.29

Results are expressed as the mean and standard deviation

**p < 0.01

***p < 0.001 by Mann–Whitney *U* test

Table 4 presents the results of multivariate logistic regression analysis of factors of vertebral fractures in patients with glucocorticoid-induced osteoporosis. Glucocorticoid treatment duration was related to disease duration. Therefore, disease duration was chosen in multivariate logistic regression analysis. Disease duration, disease duration subtracted from age, BMI, and the total hip BMD were determined to be significantly associated with vertebral fractures.

**Table 4:** Multivariate logistic regression analysis of factors of vertebral fractures in patients with glucocorticoid-induced osteoporosis

	OR	95% CI	*p* value
Disease duration	1.14	1.08–1.21	< 0.0001***
Disease duration subtracted from age	1.09	1.05–1.13	< 0.0001***
Body mass index	0.844	0.736–0.968	0.0079**
Bone mineral density			
Lumbar spine	40.3	0.85–1894	0.054
Femoral neck	36.1	0.014–92564	0.372
Total hip	6.10E-05	2.63E-08–0.137	0.009**

*OR* odds ratio; *CI* confidence interval

**p < 0.01

***p < 0.001 by multivariate logistic regression analysis

## Discussion

The present study observed significant differences related to age, BMD of the hip, disease duration, glucocorticoid treatment duration between patients with and without vertebral fractures in the glucocorticoid-induced osteoporosis group. However, no significant inter-subgroup differences were observed related to the BMD of the lumbar spine, as well as the current and cumulative doses of glucocorticoid. The result of multivariate logistic regression analysis demonstrated that the incidence of vertebral fracture was associated with disease duration, disease duration subtracted from age, BMI, and the total hip BMD.

Nonetheless, there is a mismatch between BMD data and fracture data in patients treated with glucocorticoid because of the disparity related to the alteration of bone quality. Previous studies have reported that degenerative disease of the lumbar spine and previous vertebral fracture increased BMD of the lumbar spine in elderly patients [26, 27]. Some other studies have reported that the BMD of the femur was associated with the risk factors of osteoporosis, including older age, prolonged disease duration, and low BMI in patients with rheumatological diseases [28, 29]. These results were consistent with the results of the present study. Nevertheless, hip BMD could be crucial and efficient in predicting future vertebral fractures in patients with a history of vertebral fracture.

Similar to primary osteoporosis, age, female sex, low BMI, history of falls, previous fractures, duration of menopause, and smoking are associated with fracture risk in patients on glucocorticoid. Nevertheless, a twofold increase in vertebral fracture risk is noted in patients on glucocorticoid. A large-scale study that compared patients on glucocorticoid and controls determined that the risk of hip fracture was 1.6 fold, and the risk of vertebral fracture was 2.6 fold [5]. A previous study involving 551 patients on long-term glucocorticoid treatment reported a 37% prevalence of vertebral fracture, with 48% of patients being more than 70 years old [30]. The prevalence of vertebral fracture increases with age and long-term glucocorticoid use. Several observational studies have been conducted regarding the prevalence of vertebral fracture and its relationship with the dose of glucocorticoid treatment. These studies determined that a high-dose glucocorticoid treatment, as well as a high cumulative glucocorticoid dose, were related to vertebral fracture [31, 32]. Few studies have reported that the fracture risk was more related to the current daily dose than the cumulative dose [4]. The present study demonstrated a significant difference in glucocorticoid treatment duration between patients with and without vertebral fractures. However, no significant differences related to the current and cumulative doses of glucocorticoid between groups were observed. Glucocorticoid treatment duration might be more importantly related to vertebral fractures compared with the dose of glucocorticoid. Notably, patients’ characteristics and osteoporosis treatment were not uniform among studies. Hence, these factors might influence the effects of glucocorticoid dose on vertebral fracture. Therefore, the results of these studies indicating the effects of glucocorticoid dose are debatable.

Nonetheless, our study had several limitations. First, this study was performed as a retrospective and cross-sectional study. We assessed the factors related to vertebral fracture retrospectively. Hence, further large-scale prospective studies are warranted to identify the risk factors of vertebral fracture. Second, the serum bone turnover markers and serum vitamin D levels were not measured in the present study. Third, the treatment agents used for osteoporosis and treatment duration were not uniform among the study patients. Therefore, future studies should consider assessing the risk factors of vertebral fracture in patients with uniform treatment history. Finally, underlying rheumatological diseases were not uniform in the study participants.

In conclusion, the present study indicated that disease duration, BMI, and the total hip BMD were independent risk factors of vertebral fractures in patients with glucocorticoid-induced osteoporosis. Hence, physicians should be aware that patients with longer disease duration, longer glucocorticoid treatment duration, low BMI, and low total hip BMD could be at a high risk of vertebral fracture and may require osteoporosis treatment similar to that provided for primary rheumatological diseases. Patients with rheumatological disease needing glucocorticoid should be treated with anti-resorptive agents at the early phase of glucocorticoid treatment for primary rheumatological disease. In cases with insufficient treatment effects of anti-resorptive agents, physicians should consider the treatment of teriparatide or other new therapeutic agents for osteoporosis.

## Data Availability

All data generated or analyzed during this study are included in this published article.

## Abbreviations

BMD: Bone mineral density
BMI: Body mass index
OR: Odds ratio
CI: Confidence interval
